# Electronic immunization data collection systems: application of an evaluation framework

**DOI:** 10.1186/1472-6947-14-5

**Published:** 2014-01-14

**Authors:** Christine L Heidebrecht, Jeffrey C Kwong, Michael Finkelstein, Sherman D Quan, Jennifer A Pereira, Susan Quach, Shelley L Deeks

**Affiliations:** 1 Public Health Ontario, Suite 300, 480 University Avenue, Toronto, ON M5G 1 V2, Canada; 2 Dalla Lana School of Public Health, University of Toronto, Toronto, Canada; 3 Institute for Clinical Evaluative Sciences, Toronto, Canada; 4 Department of Family and Community Medicine, University of Toronto, Toronto, Canada; 5 University Health Network, Toronto, Canada; 6 Toronto Public Health, Toronto, Canada; 7 Trillium Health Partners, Mississauga, Canada

**Keywords:** Immunization, Information systems, Data collection, Program evaluation

## Abstract

**Background:**

Evaluating the features and performance of health information systems can serve to strengthen the systems themselves as well as to guide other organizations in the process of designing and implementing surveillance tools. We adapted an evaluation framework in order to assess electronic immunization data collection systems, and applied it in two Ontario public health units.

**Methods:**

The Centers for Disease Control and Prevention’s *Guidelines for Evaluating Public Health Surveillance Systems* are broad in nature and serve as an organizational tool to guide the development of comprehensive evaluation materials. Based on these *Guidelines,* and informed by other evaluation resources and input from stakeholders in the public health community, we applied an evaluation framework to two examples of immunization data collection and examined several system attributes: simplicity, flexibility, data quality, timeliness, and acceptability. Data collection approaches included key informant interviews, logic and completeness assessments, client surveys, and on-site observations.

**Results:**

Both evaluated systems allow high-quality immunization data to be collected, analyzed, and applied in a rapid fashion. However, neither system is currently able to link to other providers’ immunization data or provincial data sources, limiting the comprehensiveness of coverage assessments. We recommended that both organizations explore possibilities for external data linkage and collaborate with other jurisdictions to promote a provincial immunization repository or data sharing platform.

**Conclusions:**

Electronic systems such as the ones described in this paper allow immunization data to be collected, analyzed, and applied in a rapid fashion, and represent the infostructure required to establish a population-based immunization registry, critical for comprehensively assessing vaccine coverage.

## Background

Each year, influenza is responsible for illness in hundreds of thousands of Canadians, and between 2,000-8,000 deaths [1]. Vaccination plays a vital role in influenza prevention, but annual revaccination is necessary due to constant viral mutation. The availability of high quality vaccine uptake and coverage data facilitates trend monitoring and programmatic response.

In Canada, the influenza vaccine is publicly funded and provided without charge to all individuals meeting high-risk criteria (e.g. elderly adults, those with chronic conditions, pregnant women, health-care workers, etc.). In some provinces, including Ontario, the vaccine is universally available to all individuals 6 months of age and older. Vaccines are administered in physician offices, healthcare facilities, public health clinics, workplace clinics, and pharmacies.

While electronic immunization information systems are becoming more widely used across Canada [2], many public health jurisdictions currently capture influenza vaccination data on paper only, or through a hybrid process in which data are initially recorded on paper and subsequently manually entered into a database or registry [3]. However, a small number of organizations have adopted systems that facilitate electronic data capture at the point of immunization. Two such systems have been developed in the province of Ontario, by Algoma Public Health (APH) and Niagara Region Public Health (NRPH) in collaboration with Niagara’s Information Technology Solutions (ITS).

Surveillance data are used for program evaluations, as well as to inform public health and clinical decision-making, research agendas and health policy development, and it is critical to ensure that the systems used to capture these data are robust and data quality is high. Periodic evaluation ensures that system processes, performance and output are comprehensively examined, and allows vulnerabilities and limitations to be identified and modifications for remediation recommended. Further, as public health authorities consider transitioning to electronic immunization information systems, existing systems serve as valuable examples. Evaluating the features and performance of these systems can serve to guide other organizations in the process of designing and implementing electronic immunization data collection systems.

Between November 2010 and March 2011 we applied an evaluation framework in a comprehensive assessment of the immunization information systems used by APH and NRPH for collecting information on influenza vaccinations given to public health clients. Although the software platform that was developed by Niagara Region has been widely used by health units across Ontario, this evaluation specifically examined its use and performance in NRPH.

## Methods

The Centers for Disease Control and Prevention’s (CDC) *Updated Guidelines for Evaluating Public Health Surveillance Systems*[4] have been extensively applied to public health information system assessments. This comprehensive tool served as the basis of our framework, designed to examine the system attributes simplicity, flexibility, data quality, timeliness, and acceptability.

### Data collection and analysis

Evaluation indicators were established based on attribute descriptions, and directed the development of specific evaluation questions, which were derived from a number of resources [4-7]. Table 1 outlines attribute definitions and corresponding data collection approaches.

**Table 1:** Attribute definitions and corresponding data collection approaches

**Attribute**	**Description**^**†**^	**Data collection approaches**
*Simplicity*	Ease of operation for vaccine delivery and support staff to collect immunization data, and for decision makers and planners to obtain the information they need for monitoring purposes.	On-site observation of data entry and extraction processes
		Key informant interviews
*Flexibility*	Capacity to accommodate modifications to reflect changing requirements and local needs.	Key informant interviews
*Data quality*	Completeness (absence of missing data elements) and validity (absence of errors in the data) of the data recorded and reported, and suitability of the data for satisfying reporting requirements. Suitability for research purposes.	Logic and completeness checks
		Client surveys
		Key informant interviews
*Timeliness*	Time required from immunization to generation of vaccine uptake estimates.	Key informant interviews
*Acceptability*	Willingness of persons and organizations to use the immunization data collection system.	Key informant interviews

^†^Based on CDC’s *Updated Guidelines for Evaluating Public Health Surveillance Systems*[4]*,* and modified for the purpose of evaluating immunization data collection systems [8].

#### ***Key informant interviews***

Semi-structured interviews were conducted with key informants in person or over the phone. Interviews encompassing questions about system processes and characteristics were conducted with one individual representing each role; shorter interviews were held with 2–3 additional system users in roles represented by multiple personnel, to obtain a broader range of feedback. Key informants were recruited with the assistance of management staff; we requested that users with an in-depth familiarity with each system be approached to complete long interviews, and asked that a larger sample of individuals be selected to complete short interviews. To increase the likelihood that they would represent a range of perspectives and experiences, rather than relying on a convenience sample, we asked management to identify this second sample based on the month in which staff members were born, although this may not have always been possible. Interview transcripts were coded by one member of the research team (CLH) and codes were corroborated by another researcher (JAP) who coded several interviews and with whom consensus was reached when discrepancies arose (this was infrequent). Codes were categorized and sorted to allow key themes from the data to emerge. The analysis was guided by our evaluation questions, but was deliberately open to allow us to observe new themes. Interviews were conducted with individuals involved with data collection, management, reporting, and decision-making in each organization.

#### ***Logic and completeness checks***

A series of logic and completeness checks were performed on 500 randomly-selected electronic records to assess the quality of core data elements. Niagara records were randomized using SQL’s random-number generator; Algoma’s records were selected using an unfiltered SQL query on a non-enumerated database. Based on Health Canada’s *Functional Standards and Minimum (Core) Data Sets for a National Immunization Registry Network*[6] and modified for the purposes of this evaluation, core data elements included: name, sex, date of birth, postal code, unique identifier (e.g. health card number), vaccine lot number, date of vaccination, anatomical site of vaccination, and dose number.

Completeness checks revealed levels of comprehensiveness among core data fields, while logic checks allowed assessment of the frequency of nonsensical information within the electronic record. Specifically, we examined the proportion of records in which:

∎ Birth date was later than date of vaccination.

∎ Date of second dose was earlier than date of first dose.

∎ Age at immunization was less than six months or greater than 110 years.

∎ Postal code did not conform to correct character order.

#### ***Client surveys***

Validity of system data was also examined using client surveys. Clients were approached by clinic staff during the 15-minute post-vaccination waiting period and asked to participate in the study. Consenting individuals completed a short survey that contained core demographic data and information about the injection site. File audits were conducted by extracting the electronic immunization file corresponding to each client form and comparing the contents of the electronic data fields against the survey data. A sample size of 158 surveys allowed detection of consistency rates of 90%, within a 5% margin of error, at a confidence level of 95%. Depending on clinic size, NRPH staff were asked to approach every 2nd or every 5th client in both the first and second half of each clinic until a set number of surveys were completed. In APH, advanced registration made it possible to randomize client numbers to identify individuals to approach for participation. A combination of low client volume, individuals failing to arrive at the appointed time, and non-registered drop-in clients required that the sampling approach be adjusted; the first person in every 15 minute time-slot was approached until the target sample size was achieved. Rates of discordance were reported and percent disagreement was calculated [9].

#### ***On-site observations***

Site visits were made to each organization’s regional headquarters. Observations of system processes, hardware, and software were recorded.

Ethics approval for all study components was granted by the University of Toronto Health Sciences Research Ethics Board, APH’s Research and Evaluation Committee, Algoma District Group Health Centre’s (GHC) Research Ethics Board, and NRPH’s Research and Evaluation Review Committee. In order to protect client data, all quantitative data quality assessments were conducted by staff in each organization; rates of discordance, incompleteness and illogical records were reported to the research team, together with descriptions of discordance. Letters of agreement between Public Health Ontario and both NRPH and APH were signed in order to allow the research team to be provided with these rates, but no individual-level data were accessed.

## Results: Algoma Public Health – EHR Influenza immunization module

Table 2 outlines the number of key informants that were interviewed, by health unit and role in the system.

**Table 2:** Key informant interviews

	**Algoma public health and group health centre**	**Niagara region public health**
Appointment^†^/registration clerks^‡^	4	2
Nurses	3	4
Data entry clerks^*^	Not applicable	2
Implementation and management staff^**^	6	8
External data recipient: ministry of health and long-term care	1	1
**Total**	**14**	**17**

^†^Group Health Centre.

^‡^Niagara Region Public Health.

^*^Paper information and consent forms are used when vaccines are administered from NRPH’s Health Bus or in the event that the system is not functioning at a clinic; the contents of these forms are manually entered into PECS.

^**^Grouped in order to protect participant confidentiality; includes IT staff, managers, coordinators, and epidemiologists.

### System description

**Figure 1:**
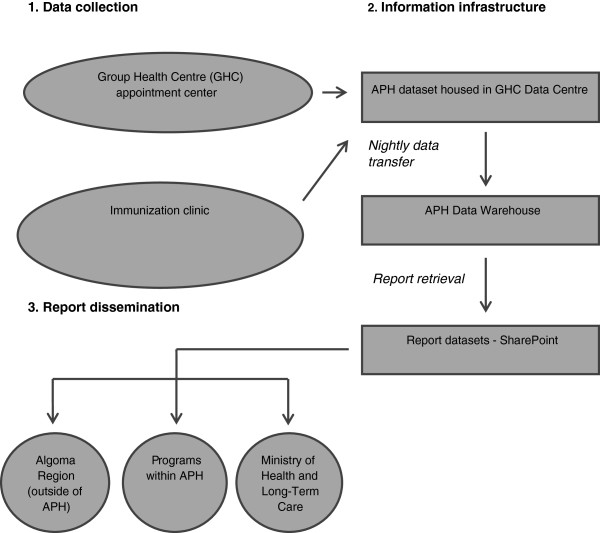
Data flow: Algoma Public Health (APH).

Born out of a collaboration with Sault Ste Marie’s Group Health Centre (GHC), which had implemented an electronic medical record (EMR) several years previously, APH’s electronic health record (EHR) was developed in 2004, sharing the infostructure – information infrastructure – that had been developed by GHC. (Representing a partnership between the Sault Ste Marie & District Group Health Association and the Algoma District Medical Group, Group Health Centre (GHC) is a health care organization comprised of a large team of health service providers, including family physicians. Over half of all family physicians in the Algoma region are part of GHC.) Client data from a range of APH programs, including influenza immunization, are captured in the EHR. Figure 1 illustrates the flow of data from data collection to report dissemination.

Immunization appointments for all APH community clinics throughout Algoma are scheduled in advance using GHC’s telephone appointment system, which interfaces with both GHC’s EMR and APH’s EHR. Clients who have not previously received GHC or APH services are assigned a unique EMR number and asked to provide name, date of birth, and phone number(s). Otherwise, the client’s identity is confirmed, and an appointment is scheduled. All vaccinations administered at APH community clinics are charted in the EHR at the point of vaccination. Each nurse at every clinic has a computer, allowing a list of all appointments to be viewed. The client’s record is retrieved by clicking on that individual’s name and their identity is confirmed; demographic information is updated or added as necessary. Medical and vaccine information are recorded on a primary data entry screen by the nurse.

Programmers are responsible for report-building; after a template has been developed, data users can access it and manipulate a range of parameters at any time to produce a report reflecting data that are current as of the previous day’s clinic(s).

### Simplicity and flexibility

Users described data input and report access as simple processes, although infrequent users may require time to refamiliarize themselves with the program. The majority of respondents reported quickly feeling comfortable with the system following training; annual refresher training is also offered. The organization’s culture of training system “super users” results in the presence of at least one nurse at every clinic with the expertise to assist users requiring support.

*It’s easy access, it’s not hard to enter data, once you know the categories… We’ve done such a good job of how to enter the data, like making the category simplistic so they can be entered easily -- we’ve done a lot of work on that. Once you get in, get the category open, get everything done it’s easy. We try to make it easy-- the system isn’t hard to do. The biggest limitation, it’s not mouse friendly; you can’t click around the mouse…For the flu, it’s fairly simple. One category, complete it, done.* [Nurse]

*Having the list of clients that are expected that day, it’s very easy to locate the person because you simply ask them what time was your appointment…So it’s very easy to locate somebody, okay. Then when you click on them, you just simply double click on their name or press “enter.” It comes up with their demographics. You verify their demographics which is usually already been done when they make the appointment…You can see up at the top just under the demographics whether or not they’ve had flu shots with the health unit or with-- at Group Health. And you simply go through all the questions, get to the bottom and do your shot and then you document right after. So it goes very, very quickly.* [Nurse]

Operational procedures are also quite straightforward. Clinic set-up requires ensuring that each nurse’s computer is authorized to access the EHR through GHC’s server and that Internet connectivity is functioning.

The nature of the relationship between APH and GHC, including the fact that data must be transferred from a GHC server to an APH server, and that access to the EHR requires logging into GHC’s server, introduces some complexity into data processing. However, in the context of this collaboration these additional steps are necessary to ensure that the integrity of the immunization data is preserved.

The EHR is very flexible. The interface through which medical history and immunization information is captured has been designed to be customizable; modifications to accommodate changing information needs or availability – for example when new vaccine lots are received – can be rapidly made without disrupting system operations or requiring external IT assistance. Likewise, report design is very accommodating to managers’ information needs. APH staff meet weekly with programmers to discuss their reporting needs, including new or modified report requests.

*For VPD* [Vaccine Preventable Diseases unit]*, the fact that I can pull reports right off the computer… It is a strength to be able to go onto a computer and find the stats for the year. Here are my stats for this vaccine. Here is this lot--, that’s a strength*. [Implementation and management staff]

Initially limited to use in permanent public health clinics across the Algoma Region, the EHR can now be accessed from authorized computers in any setting with Internet connectivity.

Influenza immunization data contained within the system are linked at the level of individual client records to all EHR data collected by other APH programs. A data sharing agreement between APH and GHC permits clinicians from both organizations to access individual-level immunization history of vaccinees who attended public clinics. However, their datasets are not linked and consequently APH cannot incorporate the vaccines administered by GHC physicians into vaccine coverage assessments. The EHR currently does not link to any immunization or other health registries outside of the Algoma Region, although the desire for this type of integration exists among data users. Specifically, APH staff would like to see linkages between the EHR and other immunization repositories in a centralized provincial immunization registry, if this were to become available in the future.

### Data quality

A number of approaches for improving data quality have been integrated into the EHR. These include:

∎ “Smart chart” technology, preventing nurses from submitting a client’s record if any fields are incomplete; this has been integrated into all mandatory fields, and logic checks have been incorporated into some of these fields.

∎ The use of drop-down menus and toggle-boxes, which minimize the number of fields requiring hand entry.

∎ Error reports examining records for incompleteness and illogical entries are run on every record, and discrepancies are corrected (errors are rare since the introduction of “smart charts”).

Management and administrators described data quality as high, and appropriate for reporting, program evaluation and research requirements. When booking client appointments, staff are trained to exhaust several search possibilities before determining that the individual does not have an electronic record in the system. It is not possible to automatically search for duplicate records; duplicates are addressed as detected.

*Very easy process. You have four ways to search before you’re going to consider entering a new patient into the system and I think it works really well. And it kind of prompts you to fill in the blanks. So it’s pretty easy. And it won’t file unless you’ve filled in the proper blanks, there’s a minimum amount of blanks you have to fill in.* [Appointment clerk]

*I like the way they can put lot numbers-- you can go into a pick list. So that makes it very* [straightforward] *-- and it’s done, you know, you’re not having to go back and do a paper record. So I can go in and see flu and I can see the tabs where people have had flu shots in the past. So that’s a quick reference for me to know that they’ve had a flu shot or -- just to see the history of how often or not that they’ve had that flu injection.* [Nurse]

Data quality was also examined through quantitative assessments. A sample of 500 client records was assessed for completeness; Table 3 illustrates the number of records in which core data elements were missing. A series of logic checks was conducted on this same sample to assess validity through exploration of nonsensical information. None of the records examined displayed any illogical patterns. The validity of system data was further examined through comparisons of a sample of 158 electronic records with client surveys. Twenty individuals who were asked to complete client surveys chose not to participate, representing approximately 11% of all individuals approached. Discordant pairs are described in Table 4.

**Table 3:** Completeness of core data elements (n = 500 records)

	**APH**			**NRPH**		
**Data element**	**Total missing**	**% missing**	**95% CI**	**Total missing**	**% missing**	**95% CI**^**‡**^
Name	0	0	--	0	0	--
Health card number	99	19.8	16.4, 23.6	26	5.2	3.4, 7.6
Sex	6	1.2	0.4, 2.6^**‡**^	0	0	--
Date of birth	0	0	--	0	0	--
Postal code	77	15.4	12.4, 18.9	25	5.0	3.2, 7.4
Vaccine lot number	0	0	--	0	0	--
Date of vaccination	0	0	--	0	0	--
Anatomical site of vaccination	0	0	--	0	0	--
Dose #	NA^†^			0	0	--

^**†**^Dose number is not a separate field because each nurse can view a client’s vaccination history and determine whether the current dose is the first or second.

^**‡**^Calculated using a Poisson distribution because the events were rare.

**Table 4:** Agreement between client surveys and electronic records

	**APH (n = 158)**			**NRPH (n = 186)**		
**Data element**	**# of discordant pairs**	**Percent disagreement**^**†**^	**95% CI**^**‡**^	**# of discordant pairs**	**Percent disagreement**^**†**^	**95% CI**^**‡**^
Last name	1	0.6	0.0, 3.5	0^*^	0	--
First name	2	1.3	0.2,4.6	3	1.7	0.3, 4.8
Sex	0	0	--	0	0	--
Date of birth	0	0	--	7	3.9	1.6, 8.1
Postal code	11	7.3	3.6, 13.0	10	5.8	2.8, 10.6
Anatomical site of vaccination	2	1.3	0.2, 4.7	1	0.6	0.0, 3.1

^**†**^Denominators varied as a result of blank fields in client surveys or electronic records.

^**‡**^Calculated using a Poisson distribution because the events were rare.

*While reflecting essentially the same name, 12 records contained surnames in which the spelling was not identical to the corresponding survey.

Both completeness and validity of EHR data were observed to be high, with the exception of health card number and postal code, neither of which are required for a client to receive an influenza immunization. We observed 7% discordance between client surveys and corresponding electronic records in the postal code field; this may be the result of client relocation rather than data entry error.

### Timeliness

Vaccine information is entered directly into each client’s electronic record at the point of immunization; transfers to APH’s Data Warehouse occur nightly, resulting in next-day availability of data reflecting vaccinations administered at any given clinic. This can be used to run clinic reports or more comprehensive assessments of vaccine uptake. Data users all indicated that this turnaround time suited their information needs, and the respondent from the Ministry of Health and Long-Term Care (MOHLTC) verified that immunization reports are received punctually.

### Acceptability

Administrative, clinical and management staff all enthusiastically indicated their commitment to the system and its successful operation. The vast majority of respondents perceived usability to be strong, data quality to be high and data quality assurance mechanisms to be effective, timing of data availability to be appropriate, and system downtime to be rare, all suggesting high user acceptability.

### Usefulness, strengths, and recommendations

The informal objectives of the EHR’s immunization module are to facilitate efficient and accurate recording and reporting of information, and to maximize clinic management and allocation of human resources. Appointment bookings are continuously monitored to ensure that staffing levels and clinic numbers and placement correspond appropriately to client requirements. Vaccine administration levels are assessed weekly to allow APH to determine where additional clinics, as well as communication to the public, could be valuable in increasing uptake. Post-campaign evaluations are conducted annually, during which the season’s rates of uptake and trends over time are considered in order to facilitate planning and promotion. These findings suggest that system objectives are being met, highlighting its usefulness. Several system strengths contribute to this usefulness:

System strengths:

∎ Timely availability of high quality data.

∎ High acceptability of the system and its data among users.

∎ Diverse range of data quality assurance mechanisms.

∎ Range of data elements available on which uptake assessments and public health planning can be based.

∎ Flexibility in report development.

∎ Advance booking system facilitates clinic and staff planning.

∎ Strong communication between system users in various roles.

To optimize system data, we recommend that APH:

∎ Promote broader data sharing with GHC physicians to increase comprehensiveness of coverage estimates.

∎ Examine possibilities for external data linkage (currently being considered).

∎ Work with other jurisdictions to advance the development of a provincial immunization repository or data sharing platform.

## Results: Niagara Region Public Health - protocol for electronic clinic systems

### System description

**Figure 2:**
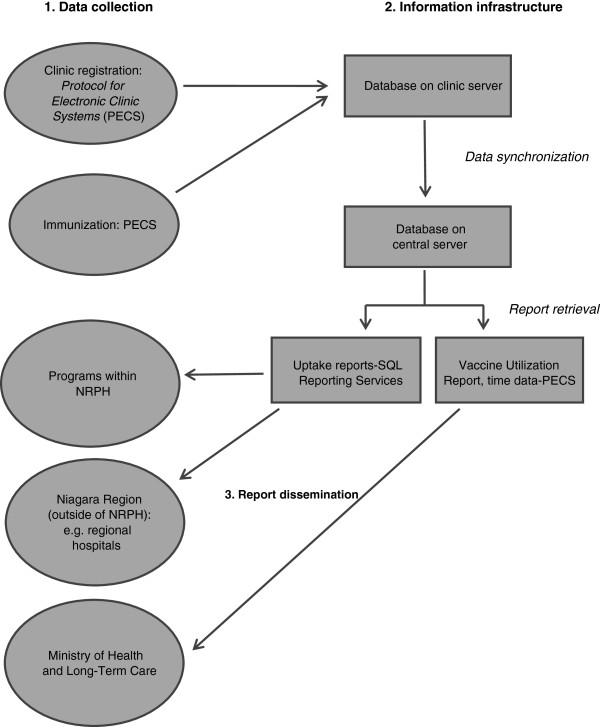
Data flow: Niagara Region Public Health (NRPH).

Developed in anticipation of the vaccine record-keeping needs that a pandemic would present, NRPH’s *Protocol for Electronic Clinic Systems* (PECS) was designed through an internal collaboration between Niagara Region’s ITS and NRPH. Figure 2 illustrates the flow of data from immunization to report dissemination. PECS currently captures influenza immunization data only.

At every public health clinic across NRPH, registration clerks and nurses are each equipped with a laptop onto which PECS has been installed. Together with a server and printer(s), these computers are linked within a local network. Upon clinic arrival, clients are registered by clerks who retrieve demographic information by swiping a health insurance card or driver’s license through a magnetic card reader, populating name, health card number, and demographic fields in the client’s profile. If neither a health card nor driver’s license is available, demographic data are captured manually. If an individual is a returning client, his/her demographic information is already available; staff verify that the information is current. Responses to medical history questions are entered into the client’s record. An immunization nurse then retrieves each individual’s electronic record from the networked database and reviews the client’s medical history before vaccine administration. Following immunization, vaccine details are entered using check-boxes and by selecting pre-populated vaccine information. Individual vaccine records created at each clinic are saved on the clinic server and, once the server has been transferred to Niagara Region’s headquarters, synchronized daily with a central database.

System data are accessed for analysis and reporting purposes through one of two mechanisms. Data required to complete the Vaccine Utilization Reports, which are submitted to MOHLTC, can be pulled directly from PECS, as well as reports reflecting clinic processing time. Other data are retrieved by running queries through a Microsoft SQL Reporting Services interface that is linked to the central immunization database. ITS personnel were involved with establishing this linkage but data users can run queries without requiring IT support.

### Simplicity and flexibility

All system users interviewed, representing administrative, clinical and management staff, described PECS as extremely user-friendly. A navigable interface and comprehensible data entry mechanisms direct nurses efficiently through registration and clinical processes. Data access for reporting purposes is also straightforward. Some nurses explained that while they felt quite comfortable with the system and found it to be intuitive, they could appreciate that it may not be straightforward for everybody, especially those who had less familiarity with electronic applications. Introductory and refresher training is provided to all nurses; at the time of this evaluation, management had recently transitioned to a model of one-on-one refresher sessions to ensure that all nurses’ individual training needs were met in a forum in which they felt comfortable asking for assistance. As in APH, NRPH trains “super users.”

*Actually, I found it very user friendly. The learning curve was pretty low… and I think the ease of use allowed me to interact with the people that much better so-- if I was constantly worried about whether something was going to work properly-- it would have been a different story but I found it exceptionally easy to use.* [Registration staff]

*When we were first given PECS to use the first year, I was very excited because I like the technology and, the cumbersomeness of the paper format that we had to use before, it reduced the amount of time substantially that we spent completing an immunization, the influenza immunization. So I didn’t find it complex at all. I found it much easier to use.* [Nurse]

Clinic logistic processes are more arduous. In order to prepare the clinic environment for PECS, a local network linking all computers and printers to a central server – on which all immunization data are stored – must be established. Following each clinic, all collected data must be transported on the server to Niagara Region headquarters and synchronized with a central database; the processes required to ensure that this synchronization is smooth and accurate were described by management and implementation staff as complex. The local network that connects all clinic computers to the server on which data are stored does not require Internet connectivity; the system can therefore operate in any environment with an electrical supply. Set-up processes are consistent across settings.

[I] *would like to find a really good, robust solution for having the clinics over the web, having them connected directly to the central database, as opposed to having that system and travelling with all that equipment and back.* [Implementation and management staff]

Report generation is a very customizable process; data analysts can design and run queries that reflect their data requirements and those of management. PECS can be quickly modified to accommodate urgent information needs, or in response to system problems without disrupting clinic operations. The system has recently been modified to allow administrative staff to change content of the health questionnaire that is asked of clients – for example, to add a symptom or allergy that was not previously applicable – although IT support is required to add or remove other data fields.

There are currently no existing linkages between PECS and other databases (for example, provincial immunization repositories or regional EMRs). Broader data integration is something that has been under consideration in NRPH and ITS and is perceived as a valuable advancement, albeit one that must be made with care in order to protect privacy and preserve data integrity.

### Data quality

Various practices and mechanisms have been integrated into PECS to improve data quality:

∎ Scanning health cards and driver’s licenses during registration, as well as the use of drop-down menus and toggle-boxes, reduces the number of fields in which hand entry errors can be made.

∎ Nurses are prevented from progressing to the next page in the application if certain fields have not been completed.

∎ Certain fields are automatically populated based on a client’s date of birth.

∎ “Masks” are placed on phone number, health card number, and date of birth fields to ensure that they fall within certain character restrictions or conform to a particular format.

∎ Pop-up windows instruct staff to verify that the record that is open corresponds to the client who is about be immunized.

∎ Scripts have recently been developed to allow duplicates to be identified and addressed following each vaccination campaign.

Management staff perceived data quality to be high, and appropriate for reporting, program evaluation, and research.

*I think having access to real time data gives a better view of what’s happening in the community. You get the demographics, I think that’s huge. I think there is enhanced ability to track. I think it cuts down on errors with paperwork. With paper forms it’s time intensive, you have to enter all of that data. It’s just so much easier to use a computerized system where you have drop-down menus that the nurses can just click. And, again, less room for error since there won’t be any issues in transcribing.* [Implementation and management staff]

Logic and completeness checks as well as client surveys were used to examine data quality in a sample of NRPH records. Table 3 illustrates the number of records, among 500 sampled, in which core data elements are missing. None of these displayed illogical patterns with respect to date of birth, dose date, or age at immunization, however, six postal codes (1.2%; 95% CI 0.4-2.6), did not conform to the correct character order. Staff compared 186 client surveys to their respective electronic records; discordant pairs are described in Table 4. We were unable to obtain information with which to calculate a survey response rate.

Completeness of PECS data is high, with the exception of health card number and postal code for which rates of incompleteness were observed to be 5%. As in APH, however, clients are not required to provide this information in order to be vaccinated by NRPH. Validity was also high in most of the elements that we examined, although we observed relatively high discordance between client surveys and electronic records for postal code and date of birth. Again, discrepancies in the postal code may be from client relocation (coupled with delays in updating health card and/or driver’s license data) rather than a data entry error. The fact that nearly 4% of the date of birth fields we examined were inconsistent with survey responses is perhaps more troubling. It is notable, however, that these dates were inconsistent in only *one* of the designations day, month, or year.

### Timeliness

Vaccine information is entered directly into each client’s electronic profile at the point of immunization; client-level data are available for review or reference immediately following vaccination. Each clinic’s data are available for analysis by the end of the day on which a clinic was held, and thus immunization reports can also be prepared in a timely manner. Data users and recipients all indicated satisfaction with this turnaround time, and immunization reports are received on time by the MOHLTC.

### Acceptability

High system acceptability was observed among all interviewed users, and was demonstrated through depictions of timely availability of data, ease of use, and the efficiency that it introduces to client interactions. Users are strongly supportive of PECS for influenza immunization data collection (to which it is currently limited), and several respondents expressed a desire to use the application for the collection of information about other vaccines.

### Usefulness, strengths, and recommendations

PECS’ objectives are to streamline client throughput, facilitate rapid availability of immunization data, and to standardize nursing and documentation practices, all of which have been achieved, illustrating the system’s usefulness. Examination of uptake by demographic characteristics supports clinic planning (locations, times, etc.), decisions regarding where promotional messages would be most useful and to whom they should be targeted, and can contribute to outbreak investigations. System data are also used to optimize clinic operations; specifically, they are used to determine appropriate ratios of registration to nursing staff, and to identify when a user may be in need of additional support (e.g. if the time that a clerk or nurse is spending with each client is substantially – and consistently – longer than other users). Many characteristics contribute to this usefulness:

System strengths:

∎ High acceptability among system users, including application of data to decision-making, and perceptions of usability.

∎ Diverse range of data quality assurance mechanisms.

∎ Range of data elements available upon which to base detailed exploration of uptake (e.g. age cohorts, postal code), as well as public health planning.

∎ Timely availability of high-quality data.

∎ Strong communication between system users in various roles.

∎ Flexibility in report development.

To enhance the impact of system data we propose the following recommendations to NRPH:

∎ Facilitate the direct transfer of clinic data to the central immunization database

∎ Consider options for expanding the application for use with other vaccines

∎ Explore possibilities for external data linkage

∎ Collaborate with other jurisdictions to promote a provincial immunization repository or data sharing platform

These are currently being considered.

## Discussion

Both systems examined in this evaluation are strong; they meet the objectives that they were developed to fulfill, producing high-quality, rapidly accessible data which are acceptable to their stakeholders. Further, respondents described a range of decisions and public health initiatives that are based on system data, including program planning and health promotion. A critical but sometimes overlooked element of a strong surveillance system is the capacity for its observations to be used in a timely fashion to inform action and the willingness of users to apply the data for this purpose. The systems described in this paper achieve a high level of performance through many shared characteristics, including the collection of a broad range of data elements and incorporation of robust quality assurance mechanisms, efficiently facilitated by electronic data collection at the point of care.

Electronic data collection practices boast many advantages over paper-based processes but consideration of the human roles behind these increasingly advanced information systems is essential to ensure that performance is high. It is evident from this assessment that time and effort have been devoted to establishing systems that function well from a data collection and management perspective, as well as a human interface perspective. The organizations’ commitment to training, including the training of “super users”, is demonstrative of this, as is the strong communication and collaboration between system users representing various roles in each organization. As a full EHR, APH’s system permits a broader range of client data – including other vaccinations – to be captured, retrieved and rapidly reported upon, and facilitates the maintenance of inclusive client records.

In both organizations, several discordant dates of birth were determined to be a result of client error during survey completion: none of the participants were infants yet all of the years of birth were recorded as the year in which the survey was administered; this occurred once in Algoma, and seven times in Niagara. It is possible that there were other instances of client error that were indiscernible, impacting rates of record agreement. Not all clients who were approached to complete surveys consented. The characteristics of individuals who chose not to participate in the client survey may have differed from those who responded, and while unlikely, these differences could have increased or decreased the probability that survey fields contained erroneous information, biasing file audit results. Further, it is conceivable that data entry errors occurred when survey data were captured prior to assessment, also resulting in discordant pairs.

Some of our observations regarding quality of data reflect limitations in the scope of their application. The absence of health card numbers in a relatively high proportion of records is a barrier to future surveillance activities requiring accurate individual-level linkage between these systems and other health records; without this or another equally pervasive unique identifier, several additional fields would need to be considered in order to be assured that corresponding records could be linked, and linkages would be probabilistic rather than deterministic. The compulsory collection of health card numbers by public health immunization providers would require a provincial decision that is not under the control of APH or NRPH. The levels of incompleteness in postal code fields, and the discordance observed between postal codes documented in surveys and system records, are problematic because they restrict the value that these data can have and could indeed result in erroneous conclusions regarding geographic distribution of vaccine uptake.

The scope of data capture for each system is currently limited to vaccinations that are administered by public health nurses; there are many vaccine providers in each jurisdiction whose data are not contained in the systems described. Consequently, neither organization is able to report complete regional vaccine coverage levels, – a problem that is common across many Canadian jurisdictions [2] and only solvable by a provincial-level registry.

Although both organizations expressed interest in being able to link their immunization data to broader datasets – provincial immunization registers and/or local EMRs – this is currently not possible, in part because the external infostructure required for provincial and other inter-jurisdictional data sharing is not yet in place. Panorama, a surveillance information system designed to monitor communicable disease outcomes [10], is currently in various stages of rollout in several Canadian provinces and will include an immunization module [2]. It is anticipated that the systems described here will be compatible with Panorama and will facilitate rapid data sharing, and in so doing will encourage other jurisdictions to adopt electronic systems of data collection, ultimately resulting in more comprehensive provincial coverage estimates.

A population-based immunization registry, into which *all* vaccines administered are entered, would provide decision-makers, planners, and evaluators with comprehensive vaccine coverage information on which program monitoring and other initiatives can be based. While Panorama has been designed as a public health information system, and thus does not currently reflect vaccines administered by physicians or other non-public health immunization providers, it is anticipated that it will become the central repository of vaccine information in each jurisdiction (province) in which it is used [10]. This will be accomplished through electronic linkages between Panorama and front-line EHR systems. In certain jurisdictions planning for this integration has begun [10], and EHRs are becoming more widely used by community providers [11] but integration between public health systems and EHRs remains a distant but promising possibility on the horizon. In the policy realm, a lack of privacy and security infrastructure is also problematic, and noncompliance with data standards in some jurisdictions is a barrier to data integration across systems [12]. Critical to the successful implementation of an integrated EHR-public health immunization system is the standardization of data content and exchange, unique identifiers, data flow, and privacy and security requirements [13]; it would be prudent for these standards to be considered now as the development of many EHRs across the country is still in its infancy and the architecture is malleable.

As Canadian jurisdictions progress towards more comprehensive linkage of immunization data, they will look to other models for direction. Systems that successfully integrate data from a diverse range of providers and documentation mechanisms that are commonly used in Canada are particularly valuable examples. Describing the care components that are monitored during a newborn’s first 48 hours of life, including immunization, an early prototype developed by Orlova et al. [14] demonstrates interoperability between EHR systems and public health systems using HL7 messaging, the standard for information exchange employed by Panorama. Integrating existing and forthcoming EHRs and local public health systems with Panorama would thus require widespread application of this standard. The Australian Childhood Immunisation Register (ACIR) contains vaccine data from a range of providers and jurisdictions [15]. This system incorporates data transmitted electronically through online notifications and provider software programs as well as paper documentation submitted by mail [16], facilitating the integration of varying levels of technology. In Canada, the Manitoba Immunization Monitoring System (MIMS) is a provincial register that reflects vaccine data collected by public health personnel and physicians, and also incorporates a range of submission mechanisms. Data entry staff capture public health data, while physician-administered immunizations are entered into the register through the province’s physician billing system [17]. In Denmark and Norway, unique identifiers assigned to each citizen – currently available at the provincial level in Canada – facilitate data linkages across numerous health record systems to populate national vaccination registers [18,19]. The development and implementation of these systems has required much communication and collaboration, and it is evident that appreciation for and commitment to collective surveillance resources among care providers and policy-makers are imperative for successful implementation.

There are system attributes in the CDC’s *Guidelines* that we either chose not to examine in this study or were unable to assess as a result of data availability. Sensitivity (the proportion of cases of a health-related event detected by a system) and positive predictive value (the proportion of detected cases that truly have the health-related event) are critical attributes for most surveillance systems but were deemed to be less important for this particular type of system where vaccination is the outcome of interest, and data are captured electronically. Because records are created at the point of registration and accessed at the point of care, a record of immunization is available for all individuals who have been vaccinated, whereas case detection of other health events varies depending on symptoms, access to services, and availability and sophistication of diagnostic tests. Similarly, because the case definition for a vaccinated individual is so straightforward it is extremely unlikely that an individual who was not vaccinated would have a record in the system; documentation is made in both systems when a vaccine has not been administered to a registered individual. While we assessed data quality of select fields through client surveys, this approach did not allow us to examine accuracy of fields critical to vaccine safety, such as lot number and adverse events and was thus a limitation of this evaluation. We had hoped to explore representativeness in order to discern how generalizable data from each electronic system could be to its respective region, but were unable to access sufficient numerator or denominator data to allow us to do so. Lastly, we assessed system stability and security and shared these findings with the participant organizations, but for purposes of brevity chose to focus on other attributes in this paper.

Since this evaluation was conducted, APH has adopted new EHR software. While some of the feedback provided by users was specific to the previous software and corresponding user interface, these comprise only one part of the overall system; the strengths and recommendations presented here have remained consistent through this transition.

## Conclusion

Our application of an evaluation framework based on the CDC’s *Guidelines* yielded a comprehensive profile of two systems which was used to draw conclusions about system usefulness and make recommendations for improved performance. Integral to the success of these evaluations were in-person observations of system processes, the opportunity to interview system users representing a range of roles, and the ability to verify electronic records using data captured through client questionnaires.

High-quality, individual-level vaccine coverage information is vital for determining levels of protection in a community, examining trends in uptake, clinic planning, and engaging in health promotion initiatives – for both influenza and other routine immunizations. Electronic systems such as the ones described in this paper allow these data to be collected, analyzed, and applied in a rapid fashion, and represent the type of infostructure required to establish a population-based immunization registry, critical for comprehensively assessing immunization coverage.

## Abbreviations

APH: Algoma Public Health; CDC: U.S. Centers for Disease Control and Prevention; EHR: Electronic health record; EMR: Electronic medical record; GHC: Group health centre; ITS: [Niagara Region] information technology solutions; NRPH: Niagara Region Public Health; PECS: Protocol for electronic clinic systems.

## Competing interests

Augmentations made to Niagara Region’s Protocol for Clinic Events System (PECS) in 2009 were supported in part by PCIRN grant funding.

## Authors’ contributions

CLH, SLD, JCK, MF, and SDQ conceived the study, modified the framework and developed data collection tools. CLH collected the data, and analyzed it, together with JAP, and SQ. CLH wrote the manuscript, and SLD, JCK, MF, SDQ, JAP, and SQ contributed to its revision. All authors read and approved the final manuscript.

## Pre-publication history

The pre-publication history for this paper can be accessed here:

http://www.biomedcentral.com/1472-6947/14/5/prepub
